# Artificial Neural Networks for Prediction of Tuberculosis Disease

**DOI:** 10.3389/fmicb.2019.00395

**Published:** 2019-03-04

**Authors:** Muhammad Tahir Khan, Aman Chandra Kaushik, Linxiang Ji, Shaukat Iqbal Malik, Sajid Ali, Dong-Qing Wei

**Affiliations:** ^1^Department of Bioinformatics and Biosciences, Capital University of Science and Technology, Islamabad, Pakistan; ^2^College of Life Sciences and Biotechnology, The State Key Laboratory of Microbial Metabolism, Shanghai Jiao Tong University, Shanghai, China; ^3^Department of Physics, Thompson Rivers University, Kamloops, BC, Canada; ^4^Provincial Tuberculosis Reference Laboratory, Hayatabad Medical Complex, Peshawar, Pakistan

**Keywords:** ANN, TB, data, diagnosis, drug resistance

## Abstract

**Background:** The global burden of tuberculosis (TB) and antibiotic resistance is attracting the attention of researchers to develop some novel and rapid diagnostic tools. Although, the conventional methods like culture are considered as the gold standard, they are time consuming in diagnostic procedure, during which there are more chances in the transmission of disease. Further, the Xpert MTB/RIF assay offers a fast diagnostic facility within 2 h, but due to low sensitivity in some sample types may lead to more serious state of the disease. The role of computer technologies is now increasing in the diagnostic procedures. Here, in the current study we have applied the artificial neural network (ANN) that predicted the TB disease based on the TB suspect data.

**Methods:** We developed an approach for prediction of TB, based on an ANN. The data was collected from the TB suspects, guardians or care takers along with samples, referred by TB units and health centers. All the samples were processed and cultured. Data was trained on 12,636 records of TB patients, collected during the years 2016 and 2017 from the provincial TB reference laboratory, Khyber Pakhtunkhwa, Pakistan. The training and test set of the suspect data were kept as 70 and 30%, respectively, followed by validation and normalization. The ANN takes the TB suspect’s information such as gender, age, HIV-status, previous TB history, sample type, and signs and symptoms for TB prediction.

**Results:** Based on TB patient data, ANN accurately predicted the *Mycobacterium tuberculosis* (MTB) positive or negative with an overall accuracy of >94%. Further, the accuracy of the test and validation were found to be >93%. This increased accuracy of ANN in the detection of TB suspected patients might be useful for early management of disease to adopt some control measures in further transmission and reduce the drug resistance burden.

**Conclusion:** ANNs algorithms may play an effective role in the early diagnosis of TB disease that might be applied as a supportive tool. Modern computer technologies should be trained in diagnostics for rapid disease management. Delays in TB diagnosis and initiation treatment may allow the emergence of new cases by transmission, causing high drug resistance in countries with a high TB burden.

## Introduction

According to the (World Health Organization [WHO], 2018), 1.7 billion people (23%) of the world’s population are estimated to have latent TB infection, indicating a risk of developing active TB during their lifetime (World Health Organization [WHO], 2018). Approximately 10.4 million incidences of TB occurred worldwide, including 5 million (56%) men, 3.5 million (34%) women and 1 million (10%) among children (WHO, 2017). Due to an increase in the world’s population, the health care units are continuously struggling to improve the standard and reduce the transmission and cost. Methods commonly used to diagnose TB include, GeneXpert assay, sputum-smear microscopy and chest radiography (Dheda et al., 2017; Ejeta et al., 2018). However, diagnosis became more complicated when the infectious agent spread to other parts of the body – this is referred to as extra pulmonary TB. All these diagnostic methods possess some limitations. Culture method is considered the gold standard for detection of the causative agent of TB, *Mycobacterium tuberculosis* (MTB) but it is time consuming in diagnosis and the chances of contamination are high (Crowle et al., 1991; Osman et al., 2010; Asgharzadeh et al., 2015). Some common issues reported from other diagnostics methods include performance issues, sputum samples from children (pediatric cases), live MTB, highly skilled medical staff for high throughput tools and high cost (Dheda et al., 2017; Pandey et al., 2017). Delay in diagnosis may lead to drug resistance, multidrug resistance (MDR), where an isolate shows resistance to two first line drugs, rifampicin and isoniazid, and extensive drug resistance (XDR) which include MDR and also show resistance to fluoroquinolones and at least one of the injectable drug (Seung et al., 2015).

In health sciences, wet lab tests can be time consuming and the chances of contamination could further lead the disease to an irreversible state. Although the Xpert MTB/RIF assay offers a fast diagnostic facility within 2 h, but due to low sensitivity in some sample types and cost may lead to more serious state of disease (Pandey et al., 2017).

In the last few decades, the researchers have collected an extensive amount of biological data in genomics, proteomics, and in some other fields of biology during the gene and protein expression analysis. To extract some meaningful information and interpret the results, high throughput computational algorithms have been developed (Fojnica et al., 2016; Dande and Samant, 2018). In bioinformatics, data mining is a process of extracting useful information deep inside of large datasets (Sebban et al., 2002; Zheng et al., 2008; Li et al., 2013). These techniques also involve artificial intelligence, statistics, machine learning, and visualization (Li et al., 2013; Dande and Samant, 2018). Such techniques are applied to expose and analyze the hidden information inside the data or sometimes also called Intelligent Data Analysis (IDA), for better prediction of results. This knowledge discovery obtained from health data has some major objectives, including diagnosis in health sciences and simulations (Mello et al., 2006; Guillet and Hamilton, 2007; Chang et al., 2012).

Traditionally implemented diagnostic methods for tuberculosis patients can be minimized with data mining approaches. National and International laboratory researchers are currently involved in developing new diagnostics, and their evaluation helps to introduce more rapid and accurate methods in the diagnosis of TB along with the evaluations of alternative algorithms for TB reference laboratories (Parsons et al., 2011).

Artificial Neural Networks (ANNs) are operated by using algorithms to interpret non-linear data, independent of sequential pattern. The networks consist of a number of smaller units called neurons, organized between the input of data and the output of results into many layers. The ANN perform and behave like biological neurons, and this behavior may be learned through a backpropagation process. In this process, the precise output of a data set is previously known as input into the network. The least mean square difference of the entire data set is minimized by the continuous comparison of output of the ANN to the known output. A good level precision is adjusted by performing complex tasks without many computing resources (Drew and Monson, 2000).

The main advantage usually provided by ANN is their capability to extract hidden linear and non-linear relationships, even in the high dimensional and complex data sets (Zhang et al., 2010). In order to ease clinical decision makers, some more rapid evaluation techniques with low costs and good precision may further support in the diagnosis of TB to give optimum time for therapy, especially in TB high burden countries. Modern methods in data mining along with some traditional methods like regression have proven to be useful for comparison of prediction power of different models. The objective behind the current study is to support physicians in diagnosis, using predictive models as a diagnostic method for TB. Here, this investigation presents the data mining methods, i.e., classification, decision tree algorithm on the TB suspect data sets with selected attributes of patients to predict the presence or absence of TB disease. This information can be applied to develop less expensive diagnostic methods, dropping drug effects, data modeling, management of health care information systems, public health, and also patient’s future prediction.

## Materials and Methods

### Ethics Statement

This study proposal was approved and permitted by Institutional Committee (Ref 30/CUST2017) and incharge and molecular biologist, Provincial Tuberculosis Research Lab (PTRL) (Ref No. 1-06-17) where individual patient names and sensitive information were removed and neither of the these have been linked with an individual TB suspect. Further, the study was also conducted according to the WHO Standards and Operational Guidance for Ethics Review of World Health Organization [WHO] (2011). Annex-3(IV)-B (13).

### Data Mining of TB Patient Data

Data in the current research was retrieved from TB control program at PTRL, Hayatabad Medical Complex Peshawar. All follow up and diagnostic patients have been included and the data of patients has been collected from guardians or care takers. The data include location, age, gender, sample type, history, HIV status. The data set contain information’s from 36 different TB units of Khyber Pakhtunkhwa (KPK). The characteristics of data is given Table 1, 2.

**Table 1:** Characteristics of TB suspects/patients received from KPK TB units.

Characteristics	No.	Characteristics	No.	Characteristics	No.
**Gender**		**Hiv status**		**Sample type**	
Female	6445	Hiv +ve	534	Sputum	11089
Male	6043	Hiv −ve	5	Pleural fluid	343
^∗^Other	148	Unknown	12097	^∗^BAL	312
Total	12636	Total	12636	Gastric aspirate	224
				^∗^CSF	172
**History**		**Age group**		Pus	124
Diagnosis	10416	0.1–14	1072	Ascetic fluid	117
Follow up	2220	15–24	3474	Tissue	104
Total	12636	25–34	2438	Urine	69
		35–44	1678	Pericardial fluid	43
**Disease type**		45–54	1690	Synovial fluid	16
Extra pulmonary	1323	55–64	1336	Lymph node	11
Pulmonary	11313	65–74	661	Total	12636
Total	12636	75–84	222	**Culture**	
		85–94	55	MTB	1809
		95–104	8	No growth	10827
		100–105	2	Total	12636
		Total	12636		

*^∗^CSF, cerebrospinal fluid; BAL, bronchoalveolar lavage; Other, she male.*

**Table 2:** Number of TB suspects received from different units of KPK province.

S.No.	Health center	Cases	S.No.	Health center	Cases
1	^∗^ATO Khyber agency	71	20	DTO Sawabi	32
2	^∗^CMH	296	21	DTO Shangla	4
3	^∗^DTO Bajawar agency	11	22	DTO Swat	6
4	DTO Bannu	8	23	Hayatabad medical complex	1903
5	DTO Buner	8	24	Khyber teaching hospital	699
6	DTO Charsadda	54	25	Kuwait hospital	21
7	DTO Chitral	5	26	Leady reading hospital	2
8	DTO D.I. Khan	8	27	Mardan medical complex	8
9	DTO Dir Lower	7	28	^∗^PMDT ATH	627
10	DTO Dir Upper	13	29	PMDT leady reading hospital	5333
11	DTO Hangu	39	30	PMDT ^∗^MMTH	1255
12	DTO Kohat	8	31	PMDT Swat	855
13	DTO Kohistan	1	32	Private	2
14	DTO Lakki	2	33	Peshawar reference lab	111
15	DTO Malakand	4	34	Rehman medical institute	66
16	DTO Mansehra	1	35	DTO Takht Nusrati	4
17	DTO Mardan	17	36	TB control Ganj	1
18	DTO Noshera	37			
19	DTO Peshawar	1117		Total	12636

*^∗^ATH, Ayub Teaching Hospital; ATO, Agency TB officers; CMH, Combined Military Hospital; DTO, district TB control officers; MMTH, Mufti Mehmood Teaching Hospital; PMDT, Programmatic Management of Drug Resistant TB.*

### Dataset Development

Suspects referred by TB units and health care centers during the years 2016 and 2017, were included. Samples were processed and cultured according the previous study (Kent and Kubica, 1985; Khan et al., 2018) and MTB negative and positive was confirmed after the culture result. Further confirmation was carried, using BD MGIT MTBc identification test (TBc ID, Ref: 245159, Becton, Dickinson), a rapid chromatographic immunoassay which detects the MTB complex antigen MPT64 secreted during culture (Arora et al., 2015). The dataset was validated by MATLAB software (Attaway, 2013). Total 12,636 inputs were used in order achieve a good output efficiency, where 70% were used as a training dataset and the remaining 30% were used as testing (Drew and Monson, 2000; Kulkarni et al., 2017). The validation observed for test dataset was about 93.71%.

### Artificial Neural Networks Approach

**FIGURE 1:**
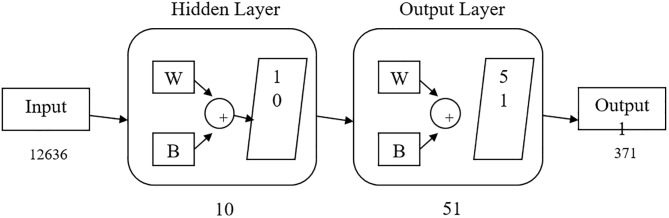
Neural network structure of MTB dataset where dataset was categorized into three categories input, hidden, and output layers. “W” represent weight parameter with layer node, “B” represent bias unit.

Artificial neural networks are nature inspired algorithms (Fojnica et al., 2016) that include input layer node, hidden layer node, and output node. Every node in a layer has one parallel node in the layer following it, thereby consequently building the stacking. Back-propagation learning algorithm is based on gradient descent search algorithms to fiddle with the correlation weight (Sollich and Krogh, 1996; Kaushik and Sahi, 2018). The output of every neuron was the aggregation of information of neurons of the prior stage multiplied by parallel weights with biased value. Input value was transformed into output with respect to activated functions shown in Figure 1.

**Step 1** – Normalization of MTB Dataset.

TB patients dataset was normalized according to proposed study (Kaushik and Sahi, 2018).

$${\text{V}}_{\text{new}}=({\text{V}}_{\text{old}}-\text{MinV})/(\text{MaxV}-\text{MinV})*({\text{D}}_{\text{max}}-{\text{D}}_{\text{min}})+{\text{D}}_{\text{min}}$$

where V_new_ represent new assessment post-normalization, V_old_ is the assessment before normalization, MinV is the variable’s minimum assessment, MaxV is the variable’s maximum estimation. Dmax and Dmin are the maximum estimation succeeding normalization and the minimum assessment subsequent to normalization, respectively.

**Step 2** – Input the data for training, the interrelated values of input and output execute for training using feed forward back propagation neural network algorithms.

**Step 3** – Set Network constraint.

**Step 4** – Calculate the neurons of output, every neuron output signal calculated using

$${\text{net}}_{\text{j}}=\sum_{\text{i}=1\sim \text{m}}{\text{w}}_{\text{ij}}{\text{x}}_{\text{i}}+{\text{b}}_{\text{j}}$$

where netj and w_ji_ are output neurons and connection weight neurons, respectively, while xi and bj are the input signal, and bias neurons. The sigmoid function or logistic function, also called the sigmoidal curve (Seggern, 2016), was used for netj and every neuron of ten hidden layers.

**Step 5** – Signal of output layers’ calculation using,

$${\text{net}}_{\text{k}}={\text{TV}}_{\text{k}}+{\text{δ}}_{\text{K}}^{\text{L}}$$

where TV_k_ is target value of output neurons and ${\text{δ}}_{\text{K}}^{\text{L}}$ is the error of neuron.

**Step 6** – Compute the error of neuron k and step 3 and step 6 were repetitive until network was congregated, and the error was computed using,

$$\text{SSE}=\sum_{\text{i}=1\sim \text{n}}{\left({\text{T}}_{\text{i}}-{\text{Y}}_{\text{i}}\right)}^{2}$$

where T_i_ is actual assessment and Y_i_ is estimated assessment.

**FIGURE 2:**
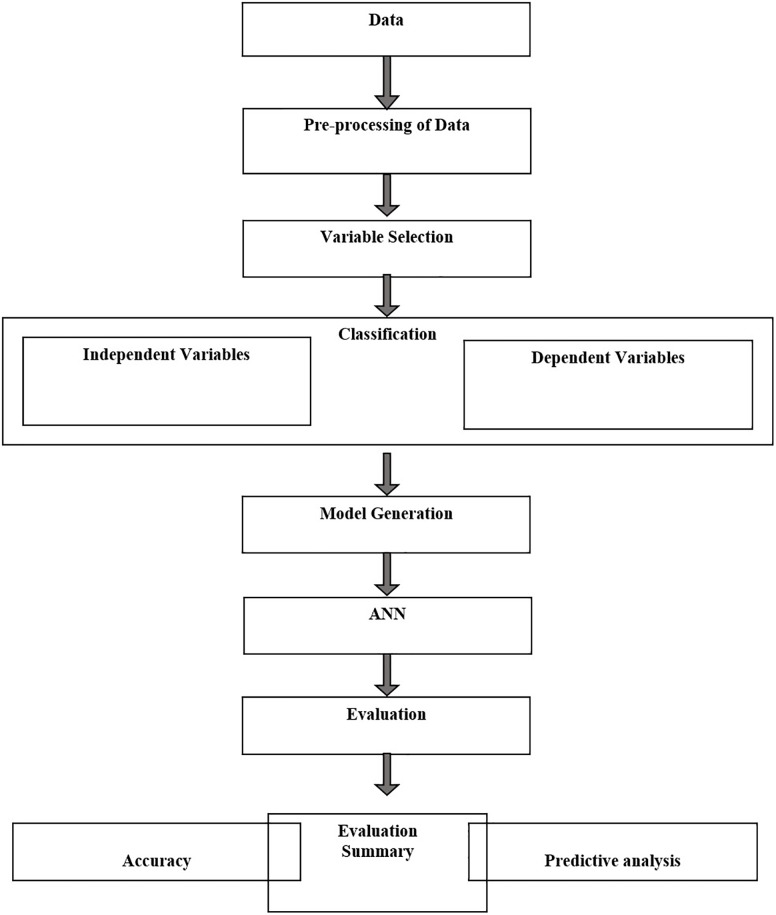
Flow chart of ANN methodology for data processing, normalization, training, testing, and prediction.

A step by step flowchart methodology has been given in Figure 1, 2.

## Results

The drug resistance and patient’s characteristics has been shown in Figure 4. MDR are very high among the population of KPK followed by other first line drug resistance. Although XDR are very few, they are very hard to treat and often take years to recover and the chances are very rare. Mono-resistant (resistant to any single drug) and poly resistant (resistant to any two or more drugs other than MDR and XDR) have been found in significant numbers. This high prevalence of drug resistance may be due to the delay type of diagnosis of some gold standard methods like culture. Owing to the current situation, we applied ANN on the patient records to find the accuracy of prediction.

Based on the ANN, the data used in the current study has 12,636 records (Table 1), where 70 and 30% were used as training and test sets, respectively. The accuracies of test and validation to predict TB based on patient data, were found at 93.90 and 93.71%, respectively. ANN-based, this algorithm accurately predicted that a suspect may have TB or not and generated the output through the hidden layer implementation. The hidden layers, learning parameters of ANN were as follows.

**Table:** 

**Number of input layer units**	**12636**
**Number of hidden layers**	10
**Number of first hidden layer units**	10
**Number of second hidden layer units**	10
**Number of output layer units**	1
**Momentum rate**	0.88
**Learning rate**	0.70
**Error after learning**	0.000050
**Learning cycle**	30,000

### The Architecture of ANN

1. Initialize the weight and parameters μ (μ = 0.01)
2. Compute the sum of the squared errors overall input F(w) = e^T^e
  Where weight of network w = [w1, w2, w3…. w_n_] and e is the error vector for the network.
3. Solve to obtain the increment of weight Δw = [J^T^ J + μI]^−1^ J^T^ e
  Where J is jacobian matrix, μ is learning rate neither μ is multiplied by decay rate β(0 < β < 1).
4. Using w+ Δw
  F(w) < F(w) then (go back to Step 2)
  W = w+ Δw
  μ = μ.β (β = 0.1) (go back to Step 2)
  ELSE
  μ = μ/β (go back to Step 2)
  END IF

The approach has been found efficient and possesses robust application for TB disease prediction with prediction accuracy. Using this approach, users can predict the active TB positively or negatively based on the patient’s data after clinical sign symptoms including, cough that lasts 3 weeks or longer and pain in the chest coughing up blood or sputum (mucus from deep inside the lungs). Other symptoms of TB disease may include; weakness or fatigue, weight loss, no appetite, chills, fever, sweating at night (CDC Tuberculosis (TB), 2019).

User input data include; age, gender (male, female, other), sample type [bone, bone marrow, bronchoalveolar lavage, cerebrospinal fluid (CSF), gastric aspirate, lung biopsy, lymph nodes, pericardial fluid, pleural fluid, synovial fluid, pus, tissue biopsy, urine, sputum], history (follow up, diagnostic), HIV status (positive, negative, unknown). The model includes 70% training and 30% test set of the entire data set (12636 records) where the validation score was achieved with an accuracy of 94%.

**FIGURE 3:**
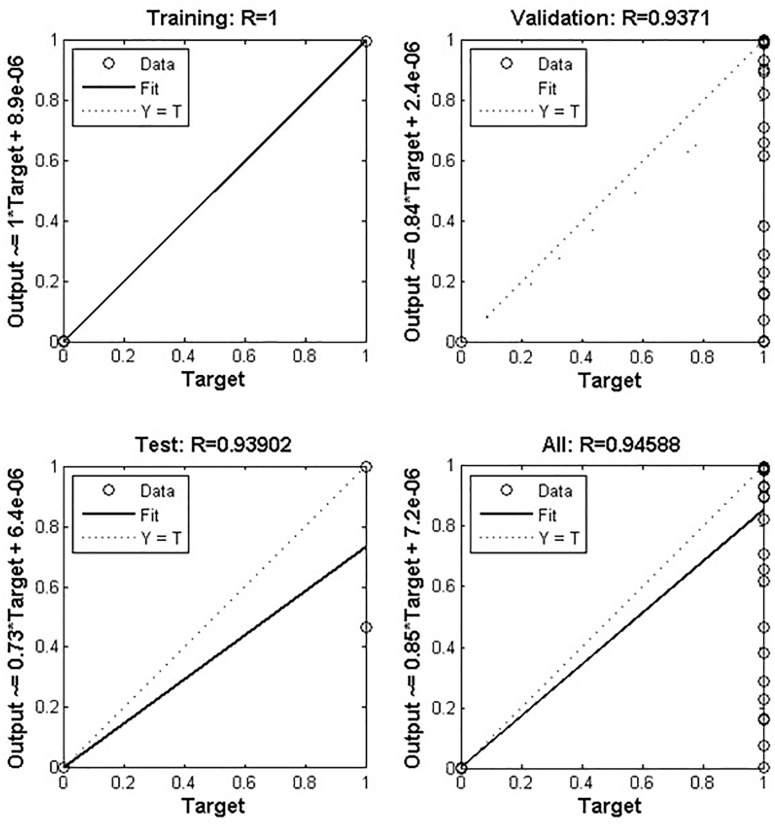
Depicts artificial neural network prediction on the basis of normalized data of MTB. Out of 12636 records, 70% was training and 30% was test set where the validation score was achieved with an accuracy of about 94%. The overall model got an accuracy of 94.58%.

The approach was written in MATLAB script where prediction accuracy was achieved as >94% based on ANN (Figure 3), dependent on dataset, where dataset input and hidden layer were categorized on two basic parameters W (weight) and B (bias unit), which contain ten sub-models and generate single output based on dataset accuracy. Users can predict their TB risk after entering their data, history, and the appearance of signs and symptoms.

## Discussion

**FIGURE 4:**
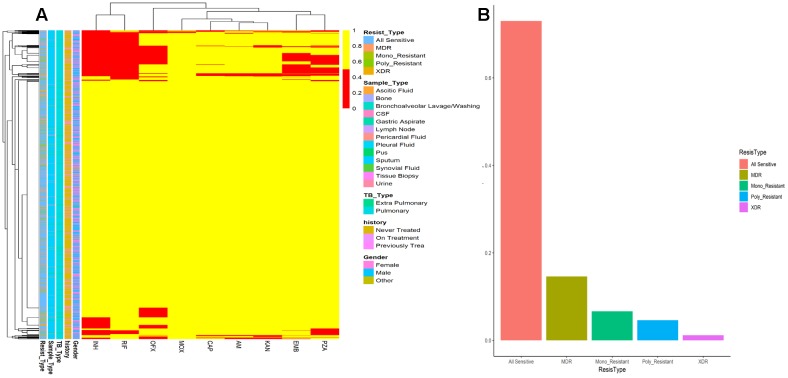
**(A)** Drug resistance among MTB isolates from Khyber Pakhtunkhwa. Frequency of all sensitive, MDR, Mono_Resistant, Poly resistant, and XDR are color coded. **(B)** Patients data and drug resistance pattern. Drug resistance is shown by red color while drug sensitive isolates are colored yellow. Resistance type (Resist_Type), Sample_Type, TB_Type, previous TB History of patients, and Gender are color coded.

Tuberculosis is a challenging disease; in spite of advanced technologies, the diagnosis is often difficult because of the nature of the disease (Dheda et al., 2017; WHO, 2017; World Health Organization [WHO], 2018). Clinical diagnosis requires standardization, where immunodiagnostic tests may help to improve sensitivity, but not in latent TB and some lack specificity (Newton et al., 2008; Elhassan et al., 2016). Xpert MTB/RIF have been saving our time to detect MTB, but decades old technologies like culture still remained the standard. Today, the battle against TB still poses one of the primary diagnostic problems in the pediatric laboratory (Dunn et al., 2016). Delay in notification and a weak coordination among TB management might be a cause to unnecessary diagnosis and treatment initiation (Yagui et al., 2006; Htun et al., 2018). Although the Xpert MTB/RIF assay offers fast diagnostic facility within 2 h, in some sample types like lymph node tissue biopsy (extrapulmonary TB) the overall sensitivity to rule out the TB is suboptimal (Creswell et al., 2014; Pandey et al., 2017; Tadesse et al., 2018). Performance was found to vary according to specimen type and acid-fast bacilli smear status. Further, the gold standard for MTB drug susceptibility testing is still culture on solid media, taking weeks to months to grow (Lin and Desmond, 2014; Dookie et al., 2018; Koch et al., 2018). Treatment is often empirical and initiated after looking at factors like past medical or social history, or the prevalence of drug resistance in that locality. These may delay the initiation of proper TB treatment that lead to drug resistance (Schaberg et al., 1996; Melchionda et al., 2013; Dookie et al., 2018; Khan et al., 2018). The high prevalence of drug resistance in TB high burden countries may delay the initiation of appropriate treatment due to culture of MTB *in vitro* which is a time consuming method. These issues should be addressed through more studies especially in TB high burden regions. We found an increased drug resistance in the recent years’ data (Figure 4) calculation, pointing toward advanced computational technologies to integrate for diagnosis in MTB prediction (Khan et al., 2018).

Modern neural networks have attained a great significance and importance in the recognition of images (Drew and Monson, 2000; Krizhevsky et al., 2012; Esteva et al., 2017; Kaushik and Sahi, 2018), speech recognition (Hinton et al., 2012), and processing of natural language (Socher et al., 2011). Medicinal researchers have started to apply these tactics in personalized clinical care. Diabetic retinopathy has also been identified through the approaches of deep neural networks (Gulshan et al., 2016) and classifying cancers of skin (Esteva et al., 2017). The applications of such approaches have also been found to be successful in computational biology and bioinformatics such as in inferring target gene expression (Chen et al., 2016), predicting RNA-binding protein sites (Zhang et al., 2016), and in identification and prediction of biomarkers for human chronological age (Putin et al., 2016). To reduce cost and time wastage, various data mining approaches may be helpful in diagnosis and on time initiation of TB therapy.

According to the WHO global TB report, 2018, India, Indonesia, China, Philippines, and Pakistan are the top five countries with 56% TB prevalence of the world. Timely TB diagnosis to reduce transmission and initiation of treatment to improve the outcomes for TB patients is essential, especially in high burden countries (Yagui et al., 2006; Dheda et al., 2017).

Classification and clustering algorithms are working efficiently with good precision in the prediction of the tuberculosis diagnosis. Presence of MTB and patient’s data may support such model up to large extents. When handling high-dimensional classification problems, different modeling approaches may be used. Earlier works have applied multivariate logistic regression (Wisnivesky et al., 2005; Solari et al., 2008), classification trees (Mello et al., 2006; Aguiar et al., 2012) and ANN (Aguiar et al., 2013; Dande and Samant, 2018) for predicting smear-negative TB.

## Conclusion

Artificial neural networks may be applied as a diagnostic tool for TB prediction and supportive in expanding the role for computer technologies in diagnostics for a rapid management of TB. Therefore, this high correlation (>94% accuracy) with the experimental result of MTB detection may help to choose optimal therapeutic regimens, especially in TB high burden countries. Delays in TB diagnosis and initiation of treatment may allow the emergence of new cases by transmission, and is one of the causes of high drug resistance in TB high burden countries.

The approach developed here may offer and support the rapid diagnosis of MTB with further additions such as drug resistance prediction in near future for better TB management.

## Data Availability

The datasets in the present study will be provided upon reasonable request to the corresponding author.

## Author Contributions

Manuscript was designed by MK, DW, SM, and LJ. ANN was written and run by AK. Data analysis and manuscript writing were carried out by MK, SA, SM, and AK. Manuscript was revised by DW, LJ, and AK.

## Conflict of Interest Statement

The authors declare that the research was conducted in the absence of any commercial or financial relationships that could be construed as a potential conflict of interest.

## Abbreviations

ANN: artificial neural network
ATH: Ayub Teaching Hospital
ATO: Agency TB officers
CSF: cerebrospinal fluid
DTO: district TB control officers
KPK: Khyber Pakhtunkhwa
MMTH: Mufti Mehmood Teaching Hospital
MTB: *Mycobacterium tuberculosis*
PMDT: Programmatic Management of Drug Resistant TB
PTRL: Provincial tuberculosis control program
TB: tuberculosis.
